# ATG7 regulates energy metabolism, differentiation and survival of Philadelphia-chromosome-positive cells

**DOI:** 10.1080/15548627.2016.1162359

**Published:** 2016-05-11

**Authors:** Maria Karvela, Pablo Baquero, Elodie M. Kuntz, Arunima Mukhopadhyay, Rebecca Mitchell, Elaine K. Allan, Edmond Chan, Kamil R. Kranc, Bruno Calabretta, Paolo Salomoni, Eyal Gottlieb, Tessa L. Holyoake, G. Vignir Helgason

**Affiliations:** a Paul O'Gorman Leukemia Research Center, College of Medical, Veterinary and Life Sciences, Institute of Cancer Sciences, University of Glasgow, Glasgow, UK; b Wolfson Wohl Cancer Research Center, College of Medical, Veterinary & Life Sciences, Institute of Cancer Sciences, University of Glasgow, UK; c Cancer Research UK, Beatson Institute, Garscube Estate, Switchback Road, Glasgow, UK; d Scottish National Blood Transfusion Service, Gartnavel General Hospital, Glasgow, UK; e Strathclyde Institute of Pharmacy and Biomedical Sciences, University of Strathclyde, Glasgow, UK; f Medical Research Council Center for Regenerative Medicine, University of Edinburgh, Edinburgh, UK; g Department of Cancer Biology, Kimmel Cancer Center, Thomas Jefferson University, Philadelphia, PA USA; h Samantha Dickson Brain Cancer Unit, UCL Cancer Institute, Paul O'Gorman Building, London, UK

**Keywords:** ATG7, autophagy, chronic myeloid leukemia, energy metabolism, erythroid differentiation, glycolysis, oxidative phosphorylation, reactive oxygen species, tyrosine kinase inhibitor

## Abstract

A major drawback of tyrosine kinase inhibitor (TKI) treatment in chronic myeloid leukemia (CML) is that primitive CML cells are able to survive TKI-mediated BCR-ABL inhibition, leading to disease persistence in patients. Investigation of strategies aiming to inhibit alternative survival pathways in CML is therefore critical. We have previously shown that a nonspecific pharmacological inhibition of autophagy potentiates TKI-induced death in Philadelphia chromosome-positive cells. Here we provide further understanding of how specific and pharmacological autophagy inhibition affects nonmitochondrial and mitochondrial energy metabolism and reactive oxygen species (ROS)-mediated differentiation of CML cells and highlight ATG7 (a critical component of the LC3 conjugation system) as a potential specific therapeutic target. By combining extra- and intracellular steady state metabolite measurements by liquid chromatography-mass spectrometry with metabolic flux assays using labeled glucose and functional assays, we demonstrate that knockdown of ATG7 results in decreased glycolysis and increased flux of labeled carbons through the mitochondrial tricarboxylic acid cycle. This leads to increased oxidative phosphorylation and mitochondrial ROS accumulation. Furthermore, following ROS accumulation, CML cells, including primary CML CD34^+^ progenitor cells, differentiate toward the erythroid lineage. Finally, ATG7 knockdown sensitizes CML progenitor cells to TKI-induced death, without affecting survival of normal cells, suggesting that specific inhibitors of ATG7 in combination with TKI would provide a novel therapeutic approach for CML patients exhibiting persistent disease.

## Introduction

Chronic myeloid leukemia (CML) arises as a consequence of a reciprocal translocation between the long arms of chromosomes 9 and 22 t(9;22)(q34;q11) that occurs within a haemopoietic stem cell (HSC).^1^ This generates the Philadelphia chromosome which carries the chimeric *BCR-ABL* gene coding for a fusion oncoprotein with constitutive tyrosine kinase activity.^2,3^ The known molecular pathogenesis of CML has facilitated the development of ABL-specific tyrosine kinase inhibitors (TKIs), such as imatinib (first generation), dasatinib, nilotinib, bosutinib (second generation) and ponatinib (third generation).^4^ TKI treatment has proven to be superior to previous forms of therapy by inducing cytogenetic and molecular responses in the majority of patients with newly diagnosed chronic phase (CP) CML.^5^ In turn, this has enabled 10% to 20% of patients to enter drug discontinuation trials^6^ and raised expectations that cure might be achievable in CML. However, with time problems of drug resistance and disease persistence have emerged in the clinic.^7,8^ It is now generally accepted that disease persistence is caused by primitive CML cells that are relatively insensitive to imatinib and other TKIs and can survive for prolonged periods of time despite complete BCR-ABL kinase inhibition.^9,10^ This implies that the majority of CML patients need to continue TKI treatment indefinitely while facing the risk of experiencing drug toxicity, TKI-resistance, relapse and/or disease progression.^4^ Further investigation into novel targetable survival pathways that are selectively active in CML cells is therefore essential.

Constitutively active BCR-ABL mimics growth factor stimulation by activating signaling pathways, such as the PI3K-AKT-MTOR pathway,^11^ that is frequently deregulated in various cancers and crucial for leukemogenesis.^12,13^ In addition, this pathway plays important roles in the regulation of HSCs, energy metabolism, mitochondrial activity, and autophagy.^14,15^ Autophagy (referring to macroautophagy) is an evolutionarily conserved catabolic process where double-membrane vesicles, termed autophagosomes, engulf cellular components and transport them to lysosomes for degradation by lysosomal hydrolases. The cargo often consists of harmful cellular material that can lead to DNA damage and genomic instability if not removed.^16^ Recycling of these intracellular components therefore promotes survival by maintaining cellular homeostasis and can also serve as an alternative source of energy during periods of metabolic stress, as well as growth factor or nutrient deprivation.^17^ Active tyrosine kinases, such as SRC and BCR-ABL, are entrapped within autophagosomes in transformed cells, suggesting that cancer cells may use autophagy to regulate and accommodate elevated levels of highly active oncogenic kinases.^18,19^ Autophagy can also lead to degradation of mitochondria (mitophagy), organelles in which pyruvate is broken down in the tricarboxylic acid (TCA) cycle to supply reducing agents (NADH and FADH_2_) for oxidative phosphorylation (OXPHOS) and ATP production.^20^ Therefore autophagy may be an important regulator of cellular metabolic capabilities.

We have previously shown that autophagy is rapidly induced following TKI treatment in CML cells and pharmacological autophagy inhibition, using the nonspecific autophagy inhibitor hydroxychloroquine (HCQ; inhibits autophagy at a late stage by preventing the fusion of autophagosomes and lysosomes), enhances the effect of TKI treatment in CML cells, including primary CD34^+^ stem or progenitor cells.^21,22^ Intrinsically linked to autophagy, energy metabolism has received significant attention over the past decade, particularly since it has become apparent that transformation from a normal cell to a cancerous one requires metabolic changes to fuel the high energy demands of cancer cells. However, whether specific autophagy inhibition affects energy metabolism or survival at the level of leukemia stem or progenitor cells is currently unknown.

Here we present novel findings regarding the mechanism through which autophagy regulates energy metabolism and reactive oxygen species (ROS)-dependent differentiation of CML cells. Firstly we show that ATG7-mediated and/or pharmacological autophagy inhibition in CML cells leads to a decrease in glycolysis and an increased flow of labeled carbons through the TCA cycle. This in turn leads to increased aerobic metabolism and mitochondrial respiration. Additionally, we show for the first time that following autophagy inhibition a metabolic switch from glycolysis to OXPHOS leads to ROS-dependent differentiation of CML cells. Finally, we show that ATG7 knockdown sensitizes CML CD34^+^ cells to TKI-induced death, without affecting non-CML CD34^+^ cells, further supporting the concept that autophagy is an important therapeutic target in CML.

## Results

### Autophagy inhibition leads to increased metabolic flux through the TCA cycle

An emerging theme is that autophagy exerts major homeostatic control on cellular metabolism.^15,23^ Cellular metabolism can be assessed by many complementary parameters, such as nutrient (i.e., glucose) uptake, metabolite secretion rates and intracellular steady-state metabolite levels. Although steady-state metabolic data is now relatively easy to generate, metabolic flux (the rate of conversion of one metabolite to another) is generally considered the critical parameter in determining the activity of a given metabolic pathway, such as glycolysis or the TCA cycle.^24^ To examine intracellular flux, heavy isotope (most frequently ^13^C) labeled nutrients (tracers), such as uniformly labeled glucose (U-^13^C_6_) are now commonly employed, which can provide critical information regarding relative pathway activities and nutrient contribution to the production of different metabolites.^25^

**Figure 1.**
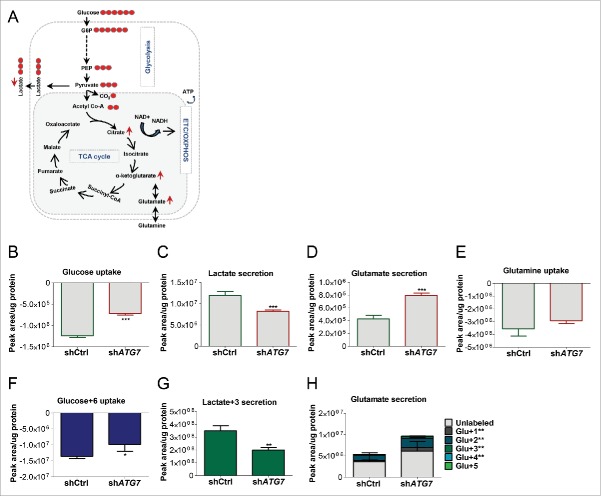
ATG7 knockdown affects extracellular metabolite levels. (A) A schematic diagram of energy metabolism in mammalian cells: During glycolysis, glucose (that contains 6 carbons) is converted into 2 molecules of the 3-carbon metabolite pyruvate via a series of intermediate metabolites. Pyruvate can then either be used to produce lactate (3-carbon molecule which is often secreted from cells) or transferred to the mitochondria for further breakdown in the TCA cycle. The TCA cycle is a series of chemical reactions, which leads to generation of energy through the oxidation of acetate (in the form of acetyl coenzyme A; acetyl Co-A) into carbon dioxide (CO_2_) and energy in the form of ATP, which is synthesized following OXPHOS in the mitochondrial ETC. U-^13^C_6_ can be used to examine flow of labeled carbons (indicated by red circles) through these pathways. During the conversion of 3-carbon pyruvate to acetyl Co-A, one carbon is converted to CO_2_ such that acetyl Co-A maintains 2 pyruvate-derived carbons (labeled) when it enters the TCA cycle. This can lead to build up of labeled carbons in TCA cycle intermediates when the pathway is activated. (B to H) sh*ATG7*-expressing K562 cells were cultured in normal medium (B to E) or in the presence of U-^13^C_6_ (F to H). Extracellular glucose (B and F), lactate (C and G), glutamate (D and H) and glutamine (E) were measured in the medium by LC-MS following 24 h culture. Note, the significance levels for metabolites with variable labeled carbons, (i.e. Glu+1 => glutamate + 1 × ^13^C) are indicated in figure legends to the right of the graph: +1, etc. corresponds to glutamate containing 1X, etc. ^13^C. Two independent experiments were performed in triplicate. *P* values: *, *P* ≤ 0.05; **, *P* ≤ 0.01; ***, *P* ≤ 0.001.

Given the essential role of ATG7 in the autophagosome completion step (*ATG7* encodes the E1-like enzyme required in both ubiquitin-like LC3 and ATG12 conjugation systems) it is a rational target to inhibit to investigate the effect of specific autophagy inhibition on different cellular processes in cancer.^26,27^ To investigate the effect of specific autophagy inhibition on energy metabolism in CML, K562 cells were transduced with a verified short-hairpin RNA (shRNA) targeting *ATG7* (sh*ATG7*)^28^ and a scrambled shRNA as a control (shCtrl). mRNA levels revealed 89% knockdown of ATG7 in sh*ATG7*-expressing cells compared with control (Fig. S1A). Following ATG7 knockdown inhibition of autophagy was verified by measuring levels of LC3B-II (lipidated form of LC3B—a marker for autophagosomes) and the autophagy substrate SQSTM1/p62^29,30^ (Fig. S1B and C). We then performed targeted metabolic analysis, focusing on glycolysis (a process where glucose is broken down to 2 molecules of pyruvate which are then either used to produce lactate or transferred to the mitochondria) and the TCA cycle (Fig. 1A). Following culture of sh*ATG7* and control cells we initially quantified the uptake and secretion of metabolites from the medium by liquid chromatography-mass spectrometry (LC-MS). This revealed a significant decrease in glucose uptake and in lactate secretion in sh*ATG7*-expressing cells, suggesting a reduction in aerobic glycolysis (Fig. 1B and C). Additionally, there was an increase in extracellular glutamate, a nonessential amino acid, which is generated from glutamine or by transamination of the TCA cycle metabolite α-ketoglutarate (Fig. 1D), without any significant changes in glutamine uptake (Fig. 1E). We then cultured cells in the presence of U-^13^C_6_ that gives an indication of flux through the metabolic pathways. Similar to Fig. 1B and C, sh*ATG7* cells consumed less U-^13^C_6_ and secreted less labeled lactate (lactate+3) compared with control cells (Fig. 1F and G), further suggesting decreased glycolytic flux. Moreover, medium from sh*ATG7* cells contained increased levels of labeled glutamate, which can only be generated following conversion of the labeled glucose via α-ketoglutarate (Fig. 1H, see legends in Fig. 1A for a description of metabolite labeling patterns). This suggested that the TCA cycle was active in autophagy-deficient cells.

**Figure 2.**
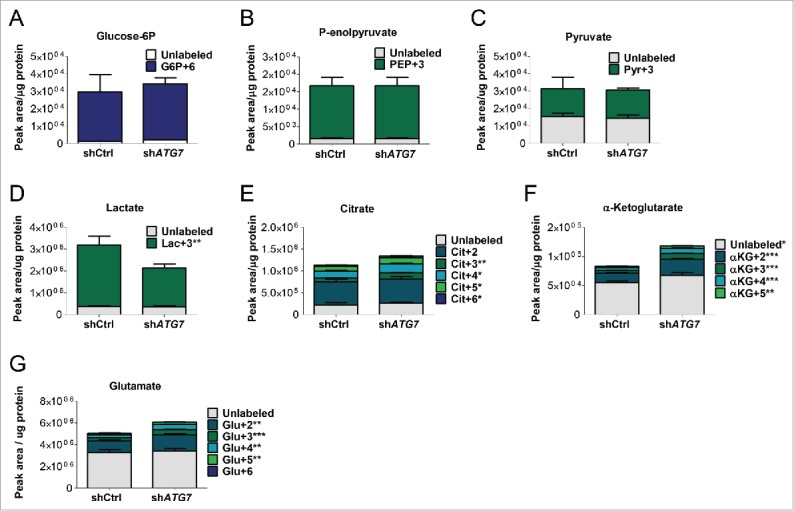
Autophagy inhibition affects intracellular metabolite levels. (A to G) Sh*ATG7*-expressing K562 cells were cultured in the presence of U-^13^C_6_ for 24 h. Following cell lysis, intercellular levels and incorporation of labeled carbons in glucose-6P (G6P) (A), P-enolpyruvate (PEP) (B), pyruvate (Pyr) (C), lactate (Lac) (D), citrate (Cit) (E), α-ketoglutarate (αKG) (F) and glutamate (Glu) (G) was measured by LC-MS. Two independent experiments were performed in triplicate. *, *P* ≤ 0.05; **, *P* ≤ 0.01; ***, *P* ≤ 0.001. +2, etc. corresponds to metabolite containing 2X, etc. ^13^C.

We next measured the levels and contribution of glucose ^13^C carbons to intracellular metabolites. While we observed no statistical change in the levels or incorporation of ^13^C into the glycolytic intermediates glucose-6-phosphate (glucose-6P), phosphoenolpyruvate (P-enolpyruvate) and pyruvate (Fig. 2A to C), there was a decrease in the levels of ^13^C incorporation into lactate (Fig. 2D). In contrast there was a significant increase in the levels of the TCA cycle intermediates, citrate and α-ketoglutarate, as well as glutamate (Fig. 2E to G). Importantly there was also an increase in incorporation of ^13^C into citrate, α-ketoglutarate and glutamate, suggesting that pyruvate is preferentially transferred to the mitochondria (instead of being converted to lactate) leading to increased metabolic flux through the TCA cycle (Fig. 2E to G, i.e. see increase in isotopologues ^13^C_4_, ^13^C_5_ and ^13^C_6_-citrate; this pattern can only be seen after a second and third round of the TCA cycle as acetyl-coenzyme A conveys only 2 ^13^C to the first round). To assess if autophagy inhibition also induced TCA activity in primary cells, CML CD34^+^ cells were treated with HCQ in medium supplemented with physiological growth factors (PGF) and U-^13^C_6_. Similar to results obtained in sh*ATG7* K562 cells, HCQ-mediated autophagy inhibition led to increased incorporation of ^13^C into citrate, α-ketoglutarate and glutamate (Fig. S2A to D) illustrating that this effect was not limited to the K562 cells. Taken together, these data suggest that autophagy regulates the balance between nonmitochondrial and mitochondrial energy pathways in CML cells.

### Autophagy inhibition leads to an increase in mitochondrial respiration

**Figure 3.**
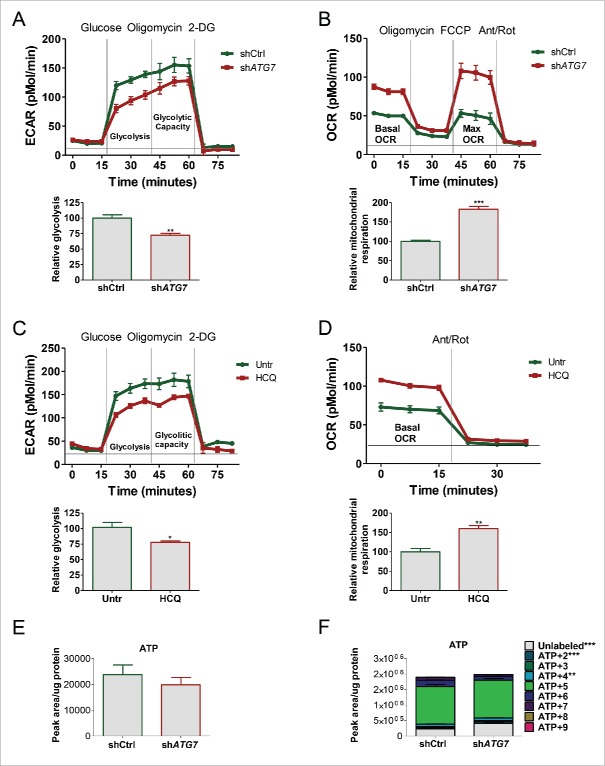
Autophagy inhibition reduces glycolysis and induces OXPHOS. ECAR (A) and OCR (B) were measured in sh*ATG7*-expressing cells using the Seahorse Extracellular Flux Analyzer. Glycolysis (relative to shCtrl-expressing cells) was calculated as “average ECAR following glucose addition minus average ECAR following inhibition of glycolysis using 2-DG.” Relative mitochondrial respiration was calculated as “basal OCR minus OCR following antimycin and rotenone (ETC inhibitors) treatment (legends for Fig. S3 provide more details). Three independent experiments were performed in quintuplicate. ECAR (C) and OCR (D) were measured in K562 cells following 24 h 10 µM HCQ treatments. Two independent experiments were performed in quintuplicate. (E and F) Sh*ATG7*-expressing K562 cells were cultured in the absence (E) or presence (F) of U-^13^C_6_ for 24 h. Following cell lysis, intercellular levels and incorporation of labeled carbons in ATP was measured by LC-MS. Two independent experiments were performed in triplicate. *, *P* ≤ 0.05; **, *P* ≤ 0.01; ***, *P* ≤ 0.001. +2, etc. corresponds to ATP containing 2X, etc. ^13^C.

In light of the results generated above by LC-MS we sought to understand more fully the effect of autophagy inhibition on energy metabolism in real time using the Seahorse Extracellular Flux Analyzer. This revealed a significant decrease in extracellular acidification rate (ECAR) in sh*ATG7*-expressing cells (Fig. 3A) in line with data in Fig. 1C and G showing decreased secretion of lactate into the culture medium. These results confirmed that autophagy deficient K562 cells are less glycolytic compared with their autophagy competent cells. Secondly, since the TCA cycle supplies reducing agents that are fed into the mitochondrial electron transport chain (ETC) to further drive ATP synthesis, we asked if upon ATG7 knockdown K562 cells increased OXPHOS to sustain energy demands and potentially compensate for the inhibition in autophagy and the reduction in aerobic glycolysis. Following measurements of mitochondrial respiration by oxygen consumption rate (OCR) we observed a significant increase in the basal, ATP-linked (basal minus oligomycin-treated cells) and maximal OCR in sh*ATG7*-expressing cells (Fig. 3B; S3A), showing that ATG7-mediated autophagy inhibition not only resulted in increased flux through the TCA cycle, but also in a parallel increase in OXPHOS. Similar increase in mitochondrial respiration was observed in the Philadelphia-chromosome-positive KCL22 cell line following ATG7 knockdown (Fig. S3B). To test if this was an ATG7-specific effect we measured glycolysis and basal OXPHOS following pharmacological autophagy inhibition. HCQ replicated the effect of ATG7 knockdown, further suggesting that autophagy regulates cellular energy metabolism in CML (Fig. 3C and D; S3C). Finally, we measured the intracellular ATP levels in sh*ATG7* and control cells (either grown in normal medium or with U-^13^C_6_). This revealed that despite the reduced glucose uptake by sh*ATG7* cells (Fig. 1B and F) the steady-state levels of ATP were unchanged and the incorporation of ^13^C carbons was comparable or slightly increased (Fig. 3E and F). This suggests that autophagy deficient CML cells may either compensate for the reduction in energy generated by glycolysis by increasing OXPHOS, or increased OXPHOS-mediated ATP generation in sh*ATG7*-expressing cells leads to decreased glucose uptake and aerobic glycolysis.

### Autophagy inhibition and elevated OXPHOS lead to an increase in mitochondrial ROS

**Figure 4.**
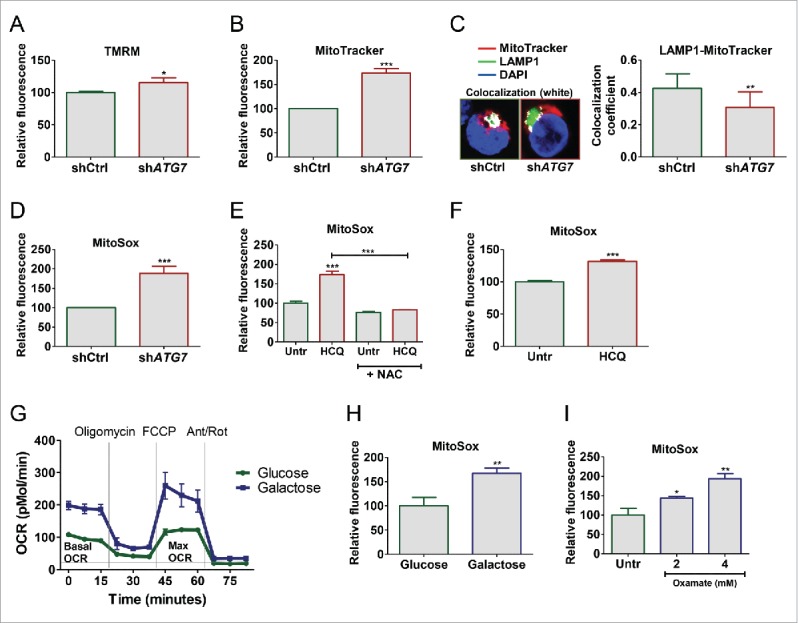
Elevated mitochondrial respiration induces ROS accumulation in CML cells. (A-B) levels of active mitochondria were measured in sh*ATG7*-expressing cells following staining of cells with TMRM (A) and MTR (B) using flow cytometry. (C) Colocalization of active mitochondria and lysosomes was measured in sh*ATG7*-expressing cells following staining of cells with MTR and LAMP1 using confocal microscopy. (D to F, as well as H and I) mitochondrial superoxide levels were measured by MitoSox staining in sh*ATG7*-expressing cells (D), K562 cells following 72 h 10 µM HCQ treatment alone and in combination with 10 nM NAC (E), CP CML CD34^+^ cells (n = 3) following 72 h 10 µM HCQ (F), K562 cells cultured in the presence of glucose (normal medium) or galactose for 72 h (H) and K562 cells treated with 2 and 4 mM oxamate for 72 h (I). Three independent experiments (A-F, H-I) were performed in duplicate. (G) OCR was measured in K562 cells cultured in the presence of 11 mM glucose or 11 nM galactose for 72 h. Three independent experiments were performed in quintuplicate. *, *P* ≤ 0.05; **, *P* ≤ 0.01; ***, *P* ≤ 0.001.

Since the principal source of ROS in the cell is the ETC we hypothesized that the increased OXPHOS in autophagy deficient CML cells would lead to an increase in mitochondrial ROS. In line with previous findings,^31-34^ after staining of K562 cells with TMRM and MTR; fluorescent dyes that are sequestered by active mitochondria dependent upon membrane potential, sh*ATG7*-expressing cells showed a significant increase in TMRM and MTR staining (Fig. 4A and B), suggesting impairment in autophagy-mediated degradation of mitochondria. Similarly, an increase in different mitochondrial proteins was also observed in sh*ATG7*-expressing cells (Fig. S4A). To further confirm that this was due to reduced clearance of active mitochondria, we costained cells with MTR and the lysosomal-associated membrane protein 1 (LAMP1). Calculations of colocalization coefficient revealed decreased overlap of LAMP1 and MTR in sh*ATG7*-expressing cells (despite an increase in both MTR and LAMP1 staining), further supporting the evidence for impaired autophagy-mediated degradation of active mitochondria in these cells (Fig. 4C; S4B). We then stained cells with MitoSox Red to measure mitochondrial superoxide levels. sh*ATG7*-expressing cells showed a significant increase in superoxide levels when compared with control cells (Fig. 4D). Treatment of K562 cells with HCQ, alone and in combination with the antioxidant N-acetyl-L-cysteine (NAC), confirmed that autophagy inhibition leads to an increase in mitochondrial ROS production (Fig. 4E; representative histogram in S4C). To assess if autophagy inhibition also induced ROS in primary cells, CML CD34^+^ cells were cultured in medium supplemented with PGF and treated with HCQ for up to 5 d (d). HCQ-mediated autophagy inhibition increased MitoSox staining indicating an increase in mitochondrial superoxide levels (Fig. 4F). Moreover, to assess if changes in energy metabolism (switch from glycolysis to OXPHOS) were sufficient to increase ROS production in CML cells we “forced” metabolic changes by altering the composition of the culture media or by inhibiting essential components of the metabolic pathway. Culturing cells in galactose (instead of glucose) as the sole sugar source forces mammalian cells to induce and rely on OXPHOS for ATP synthesis.^35,36^ To examine whether a metabolic switch from glycolysis to OXPHOS is sufficient to increase ROS production, K562 cells were cultured in normal glucose-rich medium or in the presence of galactose. This resulted in an increase in OCR and simultaneous decrease in ECAR (Fig. 4G; S4E) and increased ROS production (Fig. 4H; S4D). To further verify that an increase in OXPHOS leads to an increase in ROS production K562 cells were treated with 2 and 4 mM oxamate, an inhibitor of lactate dehydrogenase,^37^ directing pyruvate toward oxidation in the mitochondria. In line with results obtained from cells grown in galactose, oxamate-treated cells increased MitoSox staining suggesting that a metabolic switch from glycolysis to OXPHOS is sufficient to increase leakage of superoxide from the mitochondria in K562 cells (Fig. 4I; representative histogram in S4D).

### Elevated OXPHOS drives ROS-mediated differentiation of CML cells

**Figure 5.**
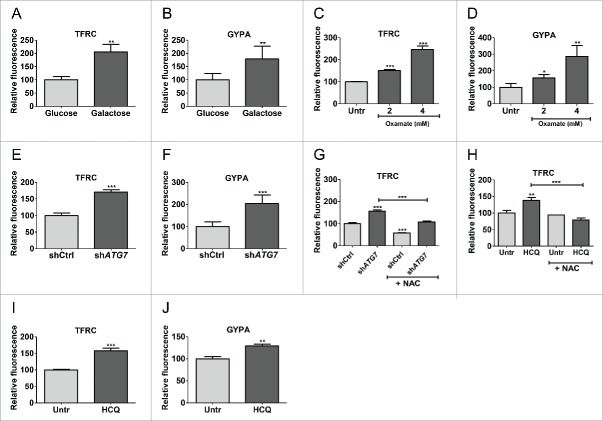
Enhanced OXPHOS drives differentiation of K562. (A to H) cellular profile was analyzed in K562 by flow cytometry for expression of TFRC (A, C, E, G and H) and GYPA (B, D and F) following 5 d culture in galactose (A and B), 5 d treatment with 2 µM and 4 µM oxamate (C and D), ATG7 knockdown alone (E and F) or +/− 10 nM NAC treatment (G) or 72 h 10 µM HCQ treatment +/− 10 nM NAC (H). Three independent experiments were performed in duplicate. (I and J) TFRC (I) and GYPA (J) levels were measured in CP CML CD34^+^ cells (n = 3) following 72 h 10 µM HCQ treatment. **, *P* ≤ 0.01; ***, *P* ≤ 0.001.

It has been suggested that high ROS correlates with myeloid differentiation of normal blood cells.^38,39^ K562 cells persist in culture in an undifferentiated state, but can spontaneously differentiate toward erythroid, granulocytic or monocytic lineages, and are therefore commonly used to measure differentiation.^40^ We therefore assessed if the increase in ROS production correlated with erythroid differentiation by culturing K562 cells in galactose followed by measurement of TFRC/CD71 (transferrin receptor) and GYPA/GlyA (glycophorin A [MNS blood group]) by flow cytometry. This revealed an increase in TFRC and GYPA expression in galactose-treated cells indicating erythroid differentiation (Fig. 5A and B). Similar results were obtained in cells treated with oxamate (Fig. 5C and D) or following ATG7 knockdown (Fig. 5E and F; representative histogram in S5A to C). Additionally, when sh*ATG7*-expressing cells were analyzed macroscopically it was evident that they had turned red, indicating increased hemoglobin production in keeping with increased TFRC and GYPA expression (Fig. S5D). Similar effect was seen following knockdown of BECN1/Beclin1, another key autophagy protein,^41^ demonstrating that the effect was not restricted to knockdown of ATG7 (Fig. S5E). To confirm that the differentiation was driven by ROS, we cultured sh*ATG*7-expressing cells with NAC followed by measurements of differentiation. This revealed that NAC partially inhibited differentiation driven by sh*ATG7* by reducing TFRC expression to similar levels as untreated control (Fig. 5G; representative histogram in S5F). Similarly, HCQ induced ROS-dependent differentiation of K562 cells (Fig. 5H).

Primary CP CML CD34^+^ cells contain a mixture of CML stem cells, multipotent and committed progenitor cells that lose CD34^+^ expression and differentiate when cultured in vitro. To assess if autophagy inhibition further promotes differentiation of primary cells, CD34^+^ CML cells were cultured in the presence of HCQ. HCQ-mediated autophagy inhibition led to an increase in TFRC and GYPA expression and a decrease in CD34^+^ expression, indicating that CML progenitor cells differentiate toward the erythroid lineage upon autophagy inhibition (Fig. 5I and J; S5G). Overall these data illustrate that autophagy inhibition results in a metabolic shift from glycolysis to OXPHOS in CML cells, leading to increased ROS production followed by differentiation.

### ATG7-mediated autophagy inhibition abrogates survival of CML CD34^+^ progenitor cells and sensitizes them to TKI-induced cell death

**Figure 6.**
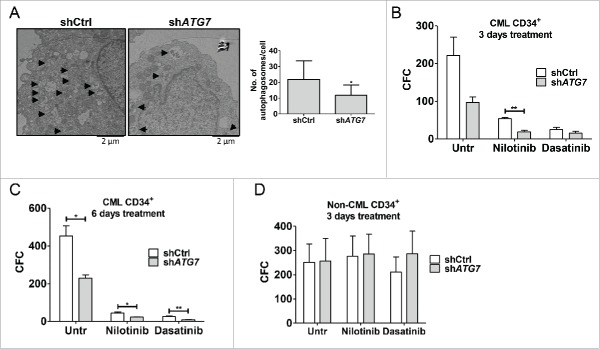
ATG7 knockdown enhances the effects of TKI treatment on survival of CP CML CD34^+^ cells. Following ATG7 knockdown CP CML CD34^+^ (A to C, n=3) or non-CML CD34^+^ cells (D, n = 3) were sorted based on GFP expression. (A) The number of autophagy-related vesicles (filled double membrane vesicles, indicated with black arrows) was quantified using electron microscopy (total of 9 cells per arm). Representative pictures are shown. (B to D) The clonogenic ability of shCtrl or sh*ATG7* CD34^+^ cells was evaluated by transferring cells to semisolid media following treatment with TKI for 3 d (B, D) or 6 d (C).

In light of these results, we sought to understand more fully the fate of patient-derived CML cells following specific autophagy inhibition. To examine whether prolonged specific autophagy inhibition affects survival of CML CD34^+^ cells, sh*ATG7* was cloned into a GFP lentiviral construct^42^ to allow immediate selection of sh*ATG7*-expressing cells. Following transduction of CP CD34^+^ cells with high titer lentivirus, autophagy inhibition was verified by western blotting on unsorted cells (Fig. S6A) and by counting the number of autophagy-related vesicles per cell by electron microscopy cells following sorting based on GFP expression (Fig. 6A). To move our results closer to the clinic and measure the effect of ATG7 knockdown, alone or in combination with TKI treatment, on expansion of CP CML CD34^+^ cells, GFP sorted cells were left untreated or treated with nilotinib or dasatinib for 3 d. This revealed that ATG7 knockdown alone inhibited expansion of CML CD34^+^ cells and combination of either nilotinib or dasatinib with ATG7 knockdown further inhibited cellular expansion (although the effect did not reach statistical significance; Fig. S6B). To measure the effect of ATG7 knockdown on survival of CML progenitor cells, GFP sorted cells were plated into methylcellulose-based medium in the presence or absence of TKIs. Colonies were counted 14 d later and revealed that stable ATG7 knockdown alone significantly reduced their numbers to a similar extent as nilotinib treatment alone (Fig. S6C). Importantly, sh*ATG7*-expressing cells were sensitized to TKI-induced death, with the difference between nilotinib alone versus nilotinib with ATG7 knockdown reaching statistical significance. The same effect was seen when GFP sorted cells were treated for 3 or 6 d in liquid culture, followed by drug washout, before being plated into colony forming cell (CFC) assay (Fig. 6B and C). This demonstrated that TKI treatment directly affected survival of progenitor cells following ATG7 knockdown.

Given the detrimental effect of *Atg7* deletion in HSC in mouse models^31^ and in an attempt to anticipate possible myelosuppression in the clinic for the combination of TKI with specific autophagy inhibition, we assessed the effect of ATG7 knockdown on primary human non-CML CD34^+^ cells. Non-CML CD34^+^ cells were transduced with sh*ATG7* lentiviruses and following sorting of GFP-positive cells the effect of ATG7 knockdown alone, or in combination with TKI treatment, on proliferation, apoptosis and CFC potential was measured. Unlike in CML cells, ATG7 knockdown had little effect on proliferation, apoptosis or CFC potential of non-CML cells, whether or not combined with TKI (Fig. 6D; S6D and E). Taken together these data indicate that TKI treatment combined with specific autophagy inhibition should achieve selectivity toward leukemic over normal progenitor cells.

## Discussion

The findings we present in this study are that following ATG7-mediated autophagy inhibition, CML cells switch from glycolysis to mitochondrial respiration, indicating that autophagy plays a role in maintaining the Warburg effect (i.e., production of energy by a high rate of aerobic glycolysis followed by lactic acid fermentation in the cytosol) in leukemic cells. The increased mitochondrial respiration was linked with an increase in mitochondrial membrane potential and ROS levels, suggesting decreased clearance of active mitochondria. This phenotype was also seen following HCQ treatment, demonstrating that the effect of ATG7 knockdown in our CML model resulted from inhibition of autophagy rather than by affecting autophagy-independent roles of ATG7. Furthermore, we showed that inhibition of autophagy and elevated OXPHOS affected differentiation of K562, KCL22 and primary CD34^+^ cells, with autophagy deficient cells differentiating toward the erythroid lineage. However, whether this would affect generation of fully enucleated red blood cells and provide a potential explanation of the severe anemia observed in mice lacking ATG7 in the hematopoietic system^33^ remains to be investigated. Additionally, given that recent findings demonstrated that BCR-ABL can be an autophagy substrate following arsenic trioxide or TKI-induced autophagy,^19,43^ it remains to be tested whether autophagy inhibition affected the levels or activity of BCR-ABL in our systems, thereby potentially promoting the enhanced ROS-mediated differentiation.

Increasing evidence suggests that ROS, which have previously been shown to be critical regulators of differentiation of normal blood cells, can act as signaling molecules.^44^ Studies in *Drosophila* have shown that increasing ROS beyond their normal levels in progenitor cells triggers differentiation by a mechanism that involves the MAPK8/JNK1-MAPK9/JNK2-MAPK10/JNK3 signaling pathway.^38^ Although the molecular mechanism by which ROS induces myeloid differentiation in our cells is not clear, it is tempting to speculate that ROS may also induce differentiation through activation of MAPK8/9/10. Indeed, increased ROS levels have previously been demonstrated to activate the MAPK8/9/10 signal transduction pathway in CML cells, as well as in unrelated systems.^44-49^

Our results also suggest that the role of autophagy in differentiation of leukemic cells may be cell type and/or disease specific since in promyelocytic leukemia, a subtype of acute myeloid leukemia, inhibition of autophagy attenuates all-trans retinoic acid-induced neutrophil differentiation,^50-52^ and pharmacological induction of autophagy potentiates all-trans retinoic acid-induced myeloid differentiation in acute myeloid leukemia.^53^ This underlines that different strategies may be required to exploit autophagy for various cancer types in the clinic.

We have previously shown that pharmacological inhibition of autophagy using chloroquine (CQ) enhances the efficacy of TKI against CML stem and progenitor cells.^21,22^ However, recent in vitro studies show that the ability of CQ to enhance chemotherapeutic responses is the same whether the cells are autophagy competent or deficient.^54^ This raises the question whether the therapeutic effects of CQ or HCQ were related to inhibition of autophagy at all. Although our previous studies in CML cell lines suggest that the effects of CQ are at least in part due to autophagy inhibition, this has not been tested in primary cells. Here we confirmed that stable and specific autophagy inhibition was detrimental for survival of primary CML. Interestingly, this dependency on ATG7 for survival was not seen following ATG7 knockdown in normal CD34^+^ cells. However, whether the difference in phenotypes following *atg7* knockout in mice^31^ vs. ATG7 knockdown in human cells reflects different roles of ATG7 depending on the environment (stem cells within the bone marrow niche in mice vs. cultured in vitro), developmental stage (*atg7* knockout mice are born autophagy-deficient) or techniques used (complete knockout vs. partial knockdown) remains to be explored. Of clinical importance, we also showed that ATG7 knockdown enhanced the effect of the second generation TKIs nilotinib and dasatinib, suggesting that the combination of specific autophagy inhibition and TKI treatment might lead to deeper response rates in CML patients with increased chance of successful drug discontinuation or even cure. Indeed, although no specific autophagy inhibitors are currently available for clinical use, with increasing understanding of the main regulators of each step of the process, some critical players appeal as attractive drug targets, such as the kinases in the ULK1 protein complex, PIK3C3, the catalytic subunit of the class III phosphatidylinositol 3-kinase, (whose yeast ortholog is Vps34) and essential “druggable” ATG proteins, such as ATG4 and ATG7 (preclinical inhibitors of some of these targets are already being developed in academia or industry).^55-59^ Therefore future examination should focus on the effect of inhibiting these proteins in a specific manner in order to find the most suitable autophagy target in CML.

## Materials and methods

### In vitro cell culture

K562 and KCL22 cells were cultured in RPMI 1640 medium, supplemented with 1% (vol/vol) penicillin/streptomycin (10,000 μg/mL/10,000U/mL, Invitrogen, 15140–122), 1% L-glutamine (Invitrogen, 25030–024), and 10% (vol/vol) fetal calf serum (Invitrogen, 10500). CP CML and non-CML CD34^+^ cells were cultured in serum-free medium comprising Iscove-modified Dulbecco medium with BIT (bovine serum albumin, insulin, transferrin; STEMCELL Technologies, 09500), 2 mmol/l glutamine, 1 mmol/l streptomycin/penicillin, 40 ng/ml low-density lipoprotein and 0.1 mmol/l 2-mercaptoethanol (Invitrogen, 21985–023). CP CML CD34^+^ cells were further supplemented with a PGF cocktail; 0.2 ng/mL SCF/GM-CSF/MIP-α, 1.0 ng/mL G-CSF/IL6 (PeproTech _EC_ Ltd, 300–07, 300–03, 300–08, 300–23 and 200–06) and 0.05 ng/mL LIF, (STEMCELL Technologies, 02642). Non-CML CD34^+^ cells were either cultured in PGF medium or further supplemented with a high-growth factor cocktail; 20 ng/mL IL3/IL6/G-CSF and 100 ng/mL Flt3L/SCF.

### Primary cell CD34 enrichment

Primary cells were obtained with informed consent from leukapheresis samples of newly diagnosed patients with CP CML or Philadelphia-chromosome-negative B cell haematological disorders. CD34^+^ cells were enriched as previously described.^21^

### Western blotting

Western blotting was performed using antibodies against p-CRKL (Cell Signaling Technology, 3181), LC3B (Cell Signaling Technology, 2775) TUBB/β-tubulin (Cell Signaling Technology, 2146), SQSTM1 (BD Biosciences, 610833), ATG7 (Epitomics, 2054–1) and Membrane Integrity WB Antibody Cocktail (Abcam, ab110414). Primary antibody detection was by enhanced chemiluminescence (GE Healthcare/Amersham, RPN2106) using a horseradish peroxidase-linked secondary antibody (Cell Signaling Technology, 7074 and 7076).

### Measurements of ECAR and OCR

ECAR and OCR were measured using the Seahorse XF96 Flux Analyzer (Seahorse Bioscience, 2100 Copenhagen, Denmark). The XF96-well plate was coated with 25 µL per well of a Cell Tak solution (22.6 ug/mL; Corning, 354240) and left for at least 30 min at room temperature. The day after K562 cells were seeded in each well in 35 µL of XF Assay Medium (Seahorse Bioscience, 100965–000) supplemented either with 2 mM glutamine for ECAR measurement or 2 mM glutamine, 11 M glucose for OCR analysis. After 30 min incubation 140 µL of medium were added to each well and the plate loaded on the analyzer after an additional 30 min incubation. The concentrations of drugs added into the cell plate during the experiment were 10 mM glucose, 10 mM 2-deoxy-D-glucose (2-DG), 1 µM oligomycin (an ATP synthase inhibitor; Sigma-Aldrich, 75351), 0.6 µM FCCP (uncouples mitochondria; Sigma-Aldrich, C2920), and 1 µM rotenone (Sigma-Aldrich, R8875) and 1 µM antimycin A (both ETC inhibitors; Sigma-Aldrich, A8674). Each time point was calculated as an average from at least 6 replicates. Error bars represent average +/− SD. Data shown are a representative experiment that was confirmed in 3 independent experiments.

### LC-MS analysis

A Thermo Scientific Exactive Orbitrap mass spectrometer was used together with a Thermo Scientific Accela HPLC system (Thermo Scientific Waltham, MA, USA). The HPLC setup consisted of a ZIC-pHILIC column (SeQuant 150×2.1 mm, 5 µm, 1.50460.0001, Merck KGaA, Darmstadt, Germany), with a ZIC-pHILIC guard column (SeQuant 20 x2.1 mm, 1.50438.0001, Merck KGaA, Darmstadt, Germany) and an initial mobile phase of 20% 20 mM ammonium carbonate, pH 9.4 (Sigma-Aldrich, 74415), and 80% acetonitrile (VWR, HiperSolv Chromanorm, 83639.320). Cell and media extracts (5 µl) were injected and metabolites were separated over a 15-min mobile phase gradient, decreasing the acetonitrile content to 20%, at a flow rate of 200 μL/min and a column temperature of 45°C. The total analysis time was 23 min. All metabolites were detected across a mass range of 75 to 1000 m/z using the Exactive mass spectrometer at a resolution of 25,000 (at 200m/z), with electrospray ionization and polarity switching to enable both positive and negative ions to be determined in the same run. Lock masses were used and the mass accuracy obtained for all metabolites was below 5 ppm. Data were acquired with Thermo Xcalibur software.

The peak areas of different metabolites were determined using Thermo TraceFinder software where metabolites were identified by the exact mass of the singly charged ion and by known retention time on the HPLC column. Commercial standards of all metabolites detected had been analyzed previously on this LC-MS system with the pHILIC column. The ^13^C labeling patterns were determined by measuring peak areas for the accurate mass of each isotopologue of many metabolites. Intracellular metabolites were normalized to cell number or protein content of the cells, measured by BCA assay.

### Flow cytometry

For measurements of mitochondrial function ATG7 knockdown, K562 cells were stained with TMRM (a cell-permeant, cationic, red-orange fluorescent dye, Life Technologies, T-668) and MitoTracker® Red CMXRos (a reduced probe that does not fluoresce until it enters live cells, where it is oxidized and sequestered in functional mitochondria and starts emitting red fluorescence, Invitrogen, M-7512). For ROS measurements ATG7 knockdown or HCQ-treated cells were washed with phosphate-buffered saline (PBS; Gibco, 14190–094, 18912–014) and incubated with 5 μM MitoSox™ Red (Invitrogen, M36008) in PBS at 37°C in the dark for 30 min. Cells were then centrifuged (400 g for 5 min) and resuspended in PBS. Fluorescence was detected by FACSVerse flow cytometry (BD Biosciences, 651155, San Jose, CA, USA). For assessment of differentiation K562 and CML CD34^+^ cells were stained with fluorochrome-conjugated mAbs directed against CD34 (BD Biosciences, 555824), TFRC (BD Biosciences, 555537) and GYPA (BD Biosciences, 561776). Data analyses were performed using FlowJo software (Tree Star).

### Cloning

The sh*ATG7* hairpin was subcloned from the pLKO.1-puro-sh*ATG7* (TRCN0000007584; Sigma-Aldrich) vector into the backbone of the pLKO.1-GFP using *Spe*I and *Nde*I restriction sites. pLKO.1-GFP backbone and sh*ATG7* insert were gel-purified by using the QIAquick Gel Extraction Kit (Qiagen, 28704) and ligated overnight at room temperature.

### Lentivirus production and transduction

pLKO.1 transfer vector containing verified shRNA specific for human *ATG7* (or nontargeting scrambled hairpin as control), pCMV-VSV-G and psPAX2 plasmids were transfected into HEK293 cells using the CaCl_2_ method as previously described.^10^ Transduction of K562 cells was performed with filtered unconcentrated virus with 70% to 95% of the cells expressing GFP after 48 h. Primary human CD34^+^ cells were transduced for 3 rounds with filtered unconcentrated lentivirus. Virus transduction reagent Transdux™ (Lonza, LV850A-1) was used to increase transduction efficiency;48 to 72 h following the third infection, cells were selected by flow cytometry based on GFP expression and cultured in medium containing PGF.

### Reagents

Imatinib and nilotinib were provided by Novartis Pharma (Basel, Switzerland). Dasatinib was provided by Bristol-Myers Squibb (Princeton, NJ, USA). Stock solution of 100 mM imatinib was prepared in sterile distilled water and stored at 4°C. Stock solutions of 10 mM nilotinib and 10 mM dasatinib were prepared in dimethyl sulphoxide (Sigma-Aldrich, A3912 100) and stored in aliquots at −20°C.

### CFC assay

Following in vitro drug treatment 5,000 cells were added to 3 mL of Methocult^™^ H4434 medium (STEMCELL Technologies, 04434). Then 1.5 mL was transferred to a 35 mm tissue culture dish in duplicate. After 14 d the number of viable colonies was counted in each dish.

### Immunofluorescence and confocal microscopy

ShCtrl and sh*ATG*7 K562 cells were incubated with 50 nM MitoTracker® Red CMXRos (Invitrogen, M-7512) for 20 min at 37°C. After washing out the stain the cells were plated on poly-l-lysine (Sigma-Aldrich, P4707) precoated multispot microscope slides (Hendley-Essex, PH-088). Cells were fixed with 3.7% formaldehyde for 15 min, and permeabilized with 0.5% Triton X-100 (Sigma-Aldrich, T8787) during 10 min. After applying blocking solution (5% BSA [Sigma-Aldrich, A3912] in PBS) the slides were incubated with LAMP1-AF488 antibody (1:100; Santa Cruz Biotechnology, 20011) overnight at 4°C (isotype control was included in every experiment). Mounting media with DAPI was used for nuclei staining and slides were analyzed using Zeiss LSM 780 confocal microscope (07745 Jena, Germany). Images were collected at 40x and 63x and the colocalization coefficient was calculated using Microscope and Imaging Software ZEN 2.1.

### Statistical analysis

Statistical analysis was performed by using unpaired Student *t* tests. A level of *P* less than or equal to 0.05 was taken to be statistically significant (*, *P*≤0.05; **, *P*≤0.01; ***, *P*≤0.001). Error bars in all figures represent standard deviation.

## Supplementary Material

Supplementary_Figures.zip

## Abbreviations

acetyl Co-A: acetyl coenzyme A
CFC: colony forming cell
CML: chronic myeloid leukemia
CP: chronic phase
CQ: chloroquine
2-DG: 2-deoxy-D-glucose
ECAR: extracellular acidification rate
ETC: electron transport chain
glucose-6P: glucose-6-phosphate
GYPA/GlyA: glycophorin A (MNS blood group)
HCQ: hydroxychloroquine
HSC: haemopoietic stem cell
LAMP1: lysosomal-associated membrane protein 1
LC-MS: liquid chromatography-mass spectrometry
MAPK: mitogen-activated protein kinase
MTR: MitoTracker Red
NAC: N-acetyl-L-cysteine
OCR: oxygen consumption rate
OXPHOS: oxidative phosphorylation
p-enolpyruvate: phosphoenolpyruvate
PBS: phosphate-buffered saline
PGF: physiological growth factors
ROS: reactive oxygen species
shCtrl: scrambled shRNA as a control
shRNA: short-hairpin RNA
TCA: tricarboxylic acid
TKI: tyrosine kinase inhibitor
TMRM: tetramethylrhodamine, methyl ester
U-^13^C_6_: uniformly labeled glucose
