# Supplementary material for: ATG7 regulates energy metabolism, differentiation and survival of Philadelphia-chromosome-positive cells
Source: Autophagy. 2016 May 11;12(6):936–48. doi: 10.1080/15548627.2016.1162359 (PMC4922442; doi:10.1080/15548627.2016.1162359)
Supplement: Supplementary_Figures.zip [file kaup-12-06-1162359-s001.zip › 2015AUTO0531R2-s03.pptx]

## Slide 1
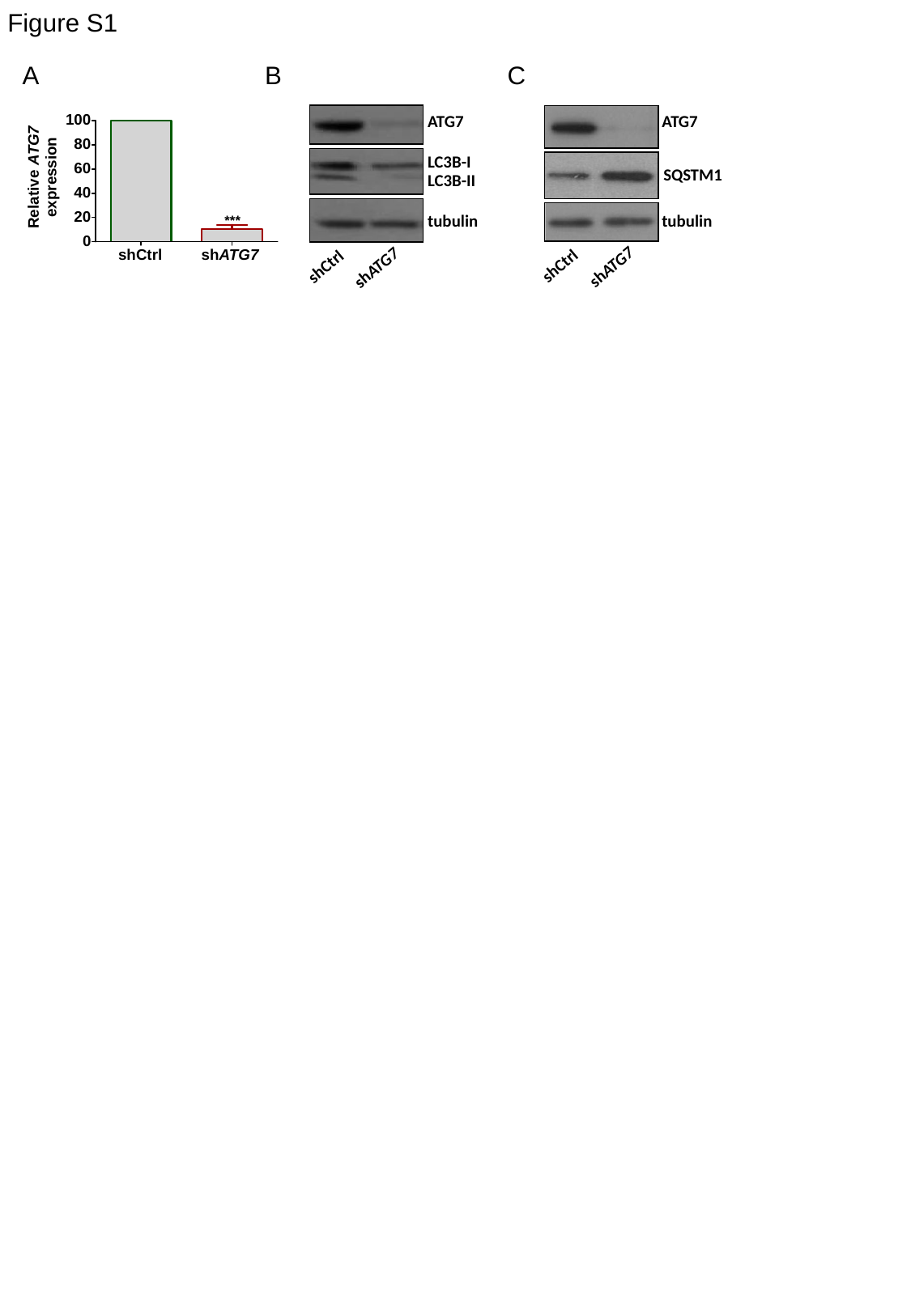

Figure S1
A
B
C
ATG7
LC3B-I
LC3B-II
tubulin
shCtrl
shATG7
ATG7
SQSTM1
tubulin
shCtrl
shATG7
shCtrl
shATG7

## Slide 2
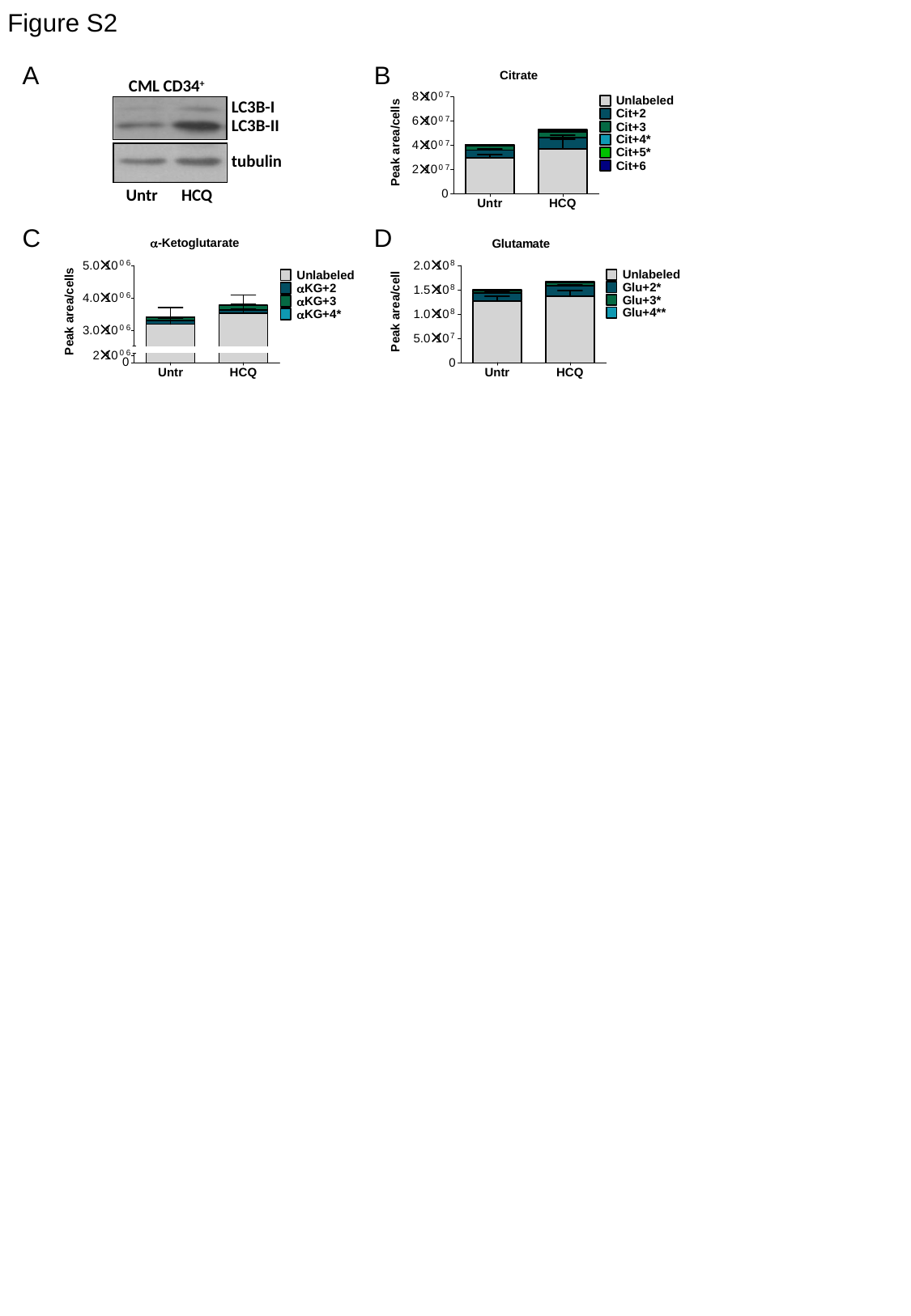

Figure S2
A
B
CML CD34+
LC3B-I
LC3B-II
tubulin
Untr
HCQ
C
D

## Slide 3
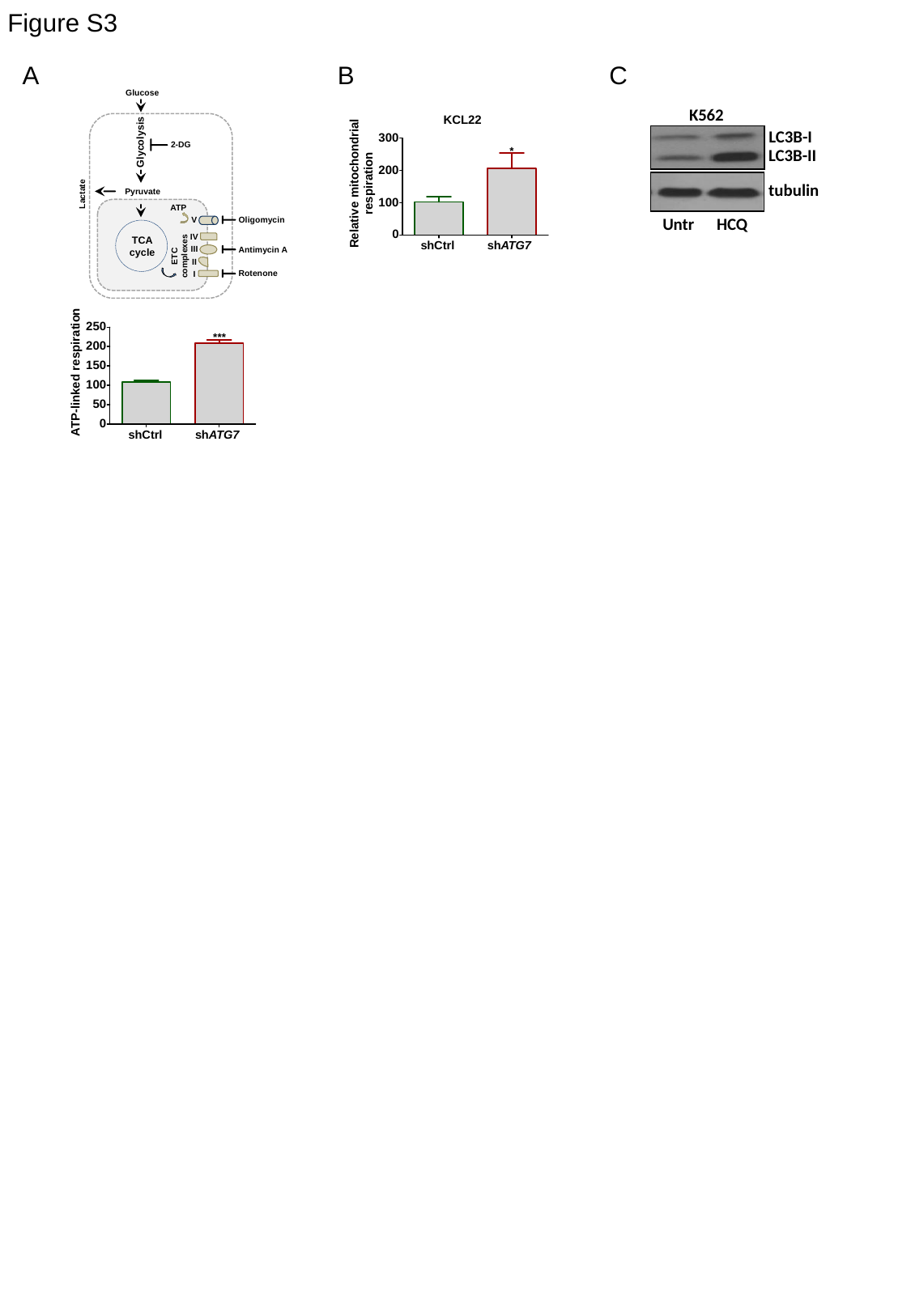

Figure S3
A
B
C
Glucose
Glycolysis
2-DG
Lactate
Pyruvate
ATP
Oligomycin
V
IV
TCA
cycle
III
Antimycin A
ETC
complexes
II
Rotenone
I
K562
LC3B-I
LC3B-II
tubulin
Untr
HCQ
shCtrl
shATG7
shCtrl
shATG7

## Slide 4
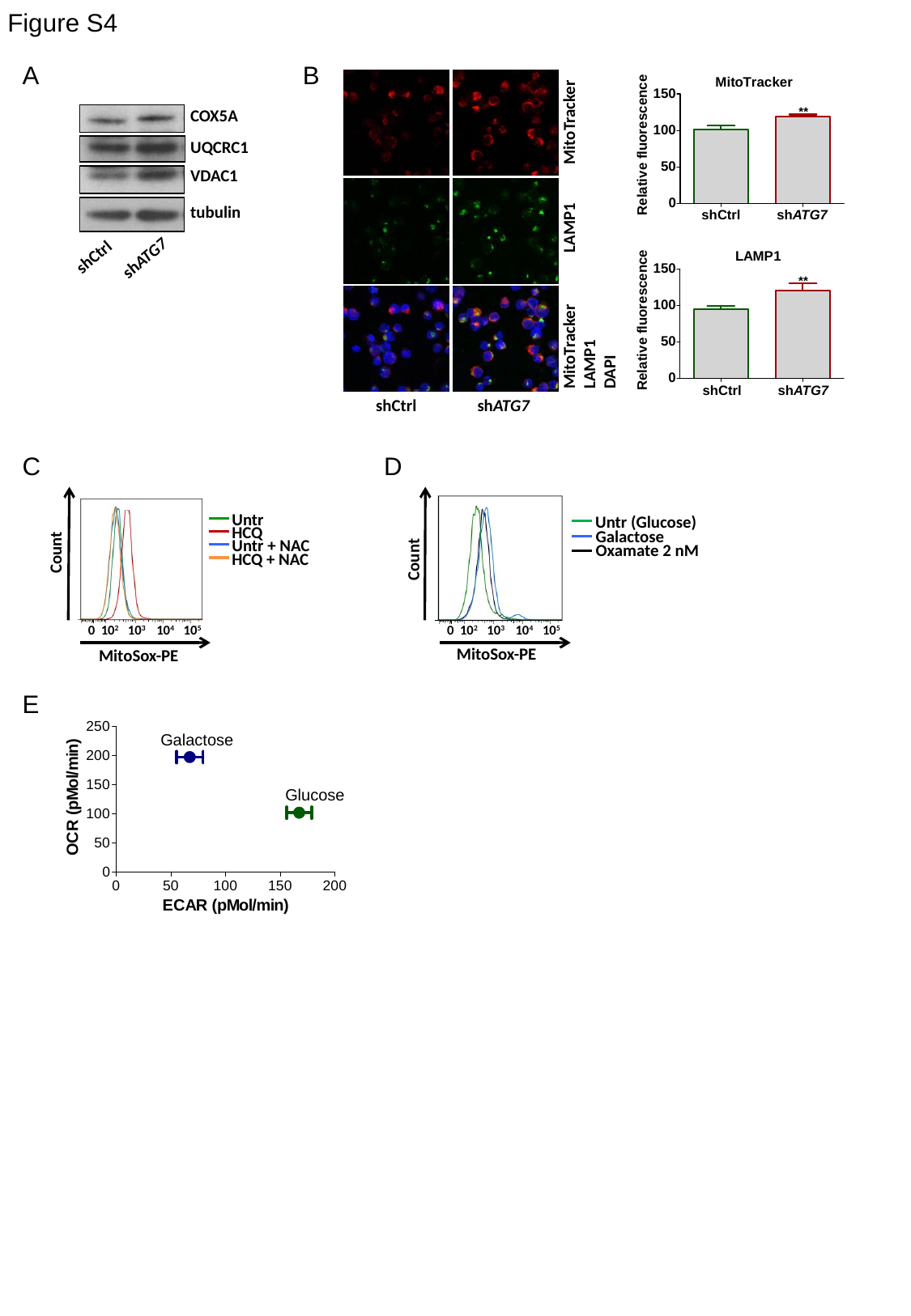

Figure S4
A
B
COX5A
UQCRC1
VDAC1
tubulin
shCtrl
shATG7
MitoTracker
shCtrl
shATG7
LAMP1
MitoTracker
LAMP1
DAPI
shCtrl
shATG7
shCtrl
shATG7
C
D
Untr
HCQ
Untr + NAC
Count
HCQ + NAC
0 102 103 104 105
MitoSox-PE
Untr (Glucose)
Galactose
Oxamate 2 nM
Count
0 102 103 104 105
MitoSox-PE
E

## Slide 5
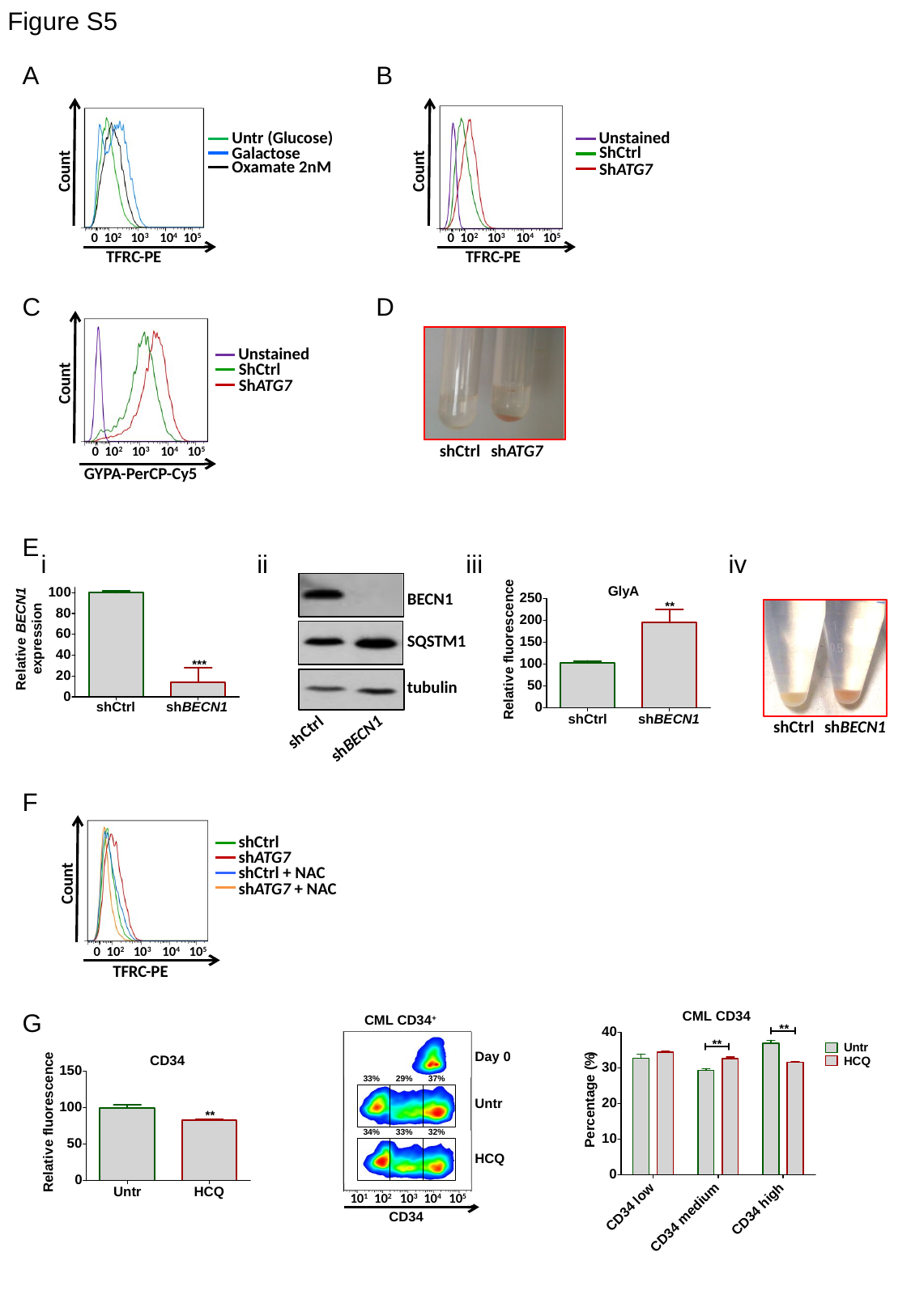

Figure S5
A
B
Galactose
Oxamate 2nM
0 102 103 104 105
TFRC-PE
Unstained
ShCtrl
Count
ShATG7
0 102 103 104 105
TFRC-PE
Untr (Glucose)
Count
C
D
Unstained
ShCtrl
Count
ShATG7
0 102 103 104 105
GYPA-PerCP-Cy5
shCtrl
shATG7
E
i
ii
iii
iv
BECN1
SQSTM1
tubulin
shCtrl
shBECN1
shCtrl
shBECN1
shCtrl
shBECN1
shCtrl
shBECN1
F
shCtrl
shATG7
shCtrl + NAC
Count
shATG7 + NAC
0 102 103 104 105
TFRC-PE
G
CML CD34+
33%
29%
37%
34%
33%
32%
CD34
Day 0
Untr
HCQ
101 102 103 104 105

## Slide 6
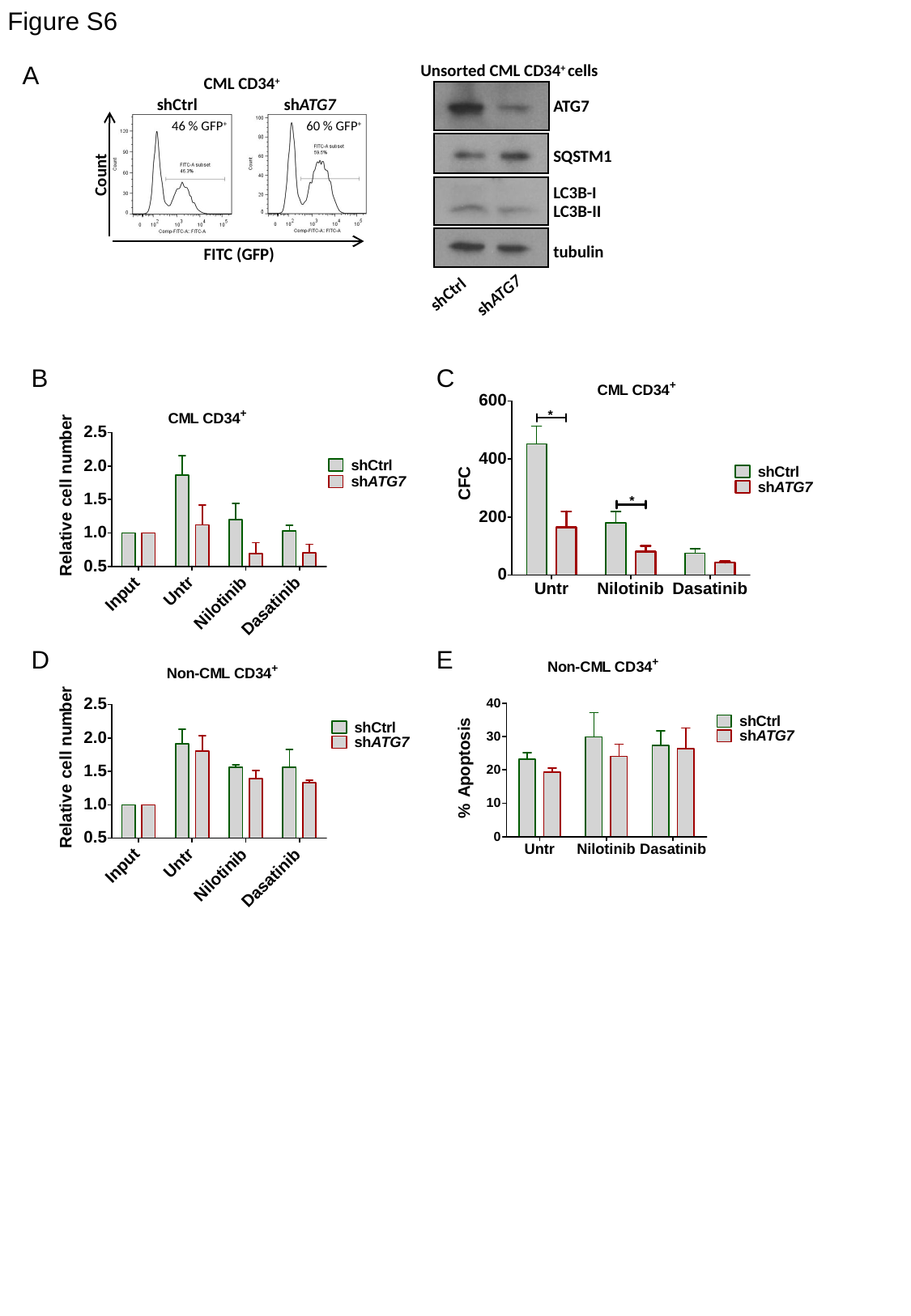

Figure S6
A
Unsorted CML CD34+ cells
ATG7
SQSTM1
LC3B-I
LC3B-II
tubulin
shCtrl
shATG7
CML CD34+
shCtrl
shATG7
46 % GFP+
60 % GFP+
Count
FITC (GFP)
B
C
D
E
